# Supplementary material for: Double-Bilayer polar nanoregions and Mn antisites in (Ca, Sr)3Mn2O7
Source: Nat Commun. 2022 Aug 22;13:4927. doi: 10.1038/s41467-022-32090-w (PMC9395386; doi:10.1038/s41467-022-32090-w)
Supplement: Supplementary file 1 — Supplementary Results for Double-Bilayer Polar Nanoregions and Mn Antisites in (Ca, Sr)3Mn2O7 [file 41467_2022_32090_MOESM1_ESM.docx]

Supplementary Information for

Double-Bilayer Polar Nanoregions and Mn Antisites in (Ca, Sr)_3_Mn_2_O_7_

Leixin Miao^1^, Kishwar-E Hasin^2^, Parivash Moradifar^1,3^, Debangshu Mukherjee^4^, Ke Wang^5^, Sang-Wook Cheong^6^, Elizabeth A. Nowadnick^2^ & Nasim Alem^1,5*^

^1^ Department of Materials Science and Engineering, The Pennsylvania State University, University Park, PA, 16802, USA

^2^ Department of Materials Science and Engineering, University of California, Merced, CA, 95343, USA

^3^ Department of Materials Science and Engineering, Stanford University, Stanford, California 94305, USA

^4^ Computational Sciences & Engineering Division, Oak Ridge National Laboratory, Oak Ridge, TN, 37830, USA

^5^ Materials Research Institute, The Pennsylvania State University, University Park, PA, 16802, USA

^6^ Rutgers Center for Emergent Materials and Department of Physics and Astronomy, Rutgers University, Piscataway, New Jersey, 08854, USA


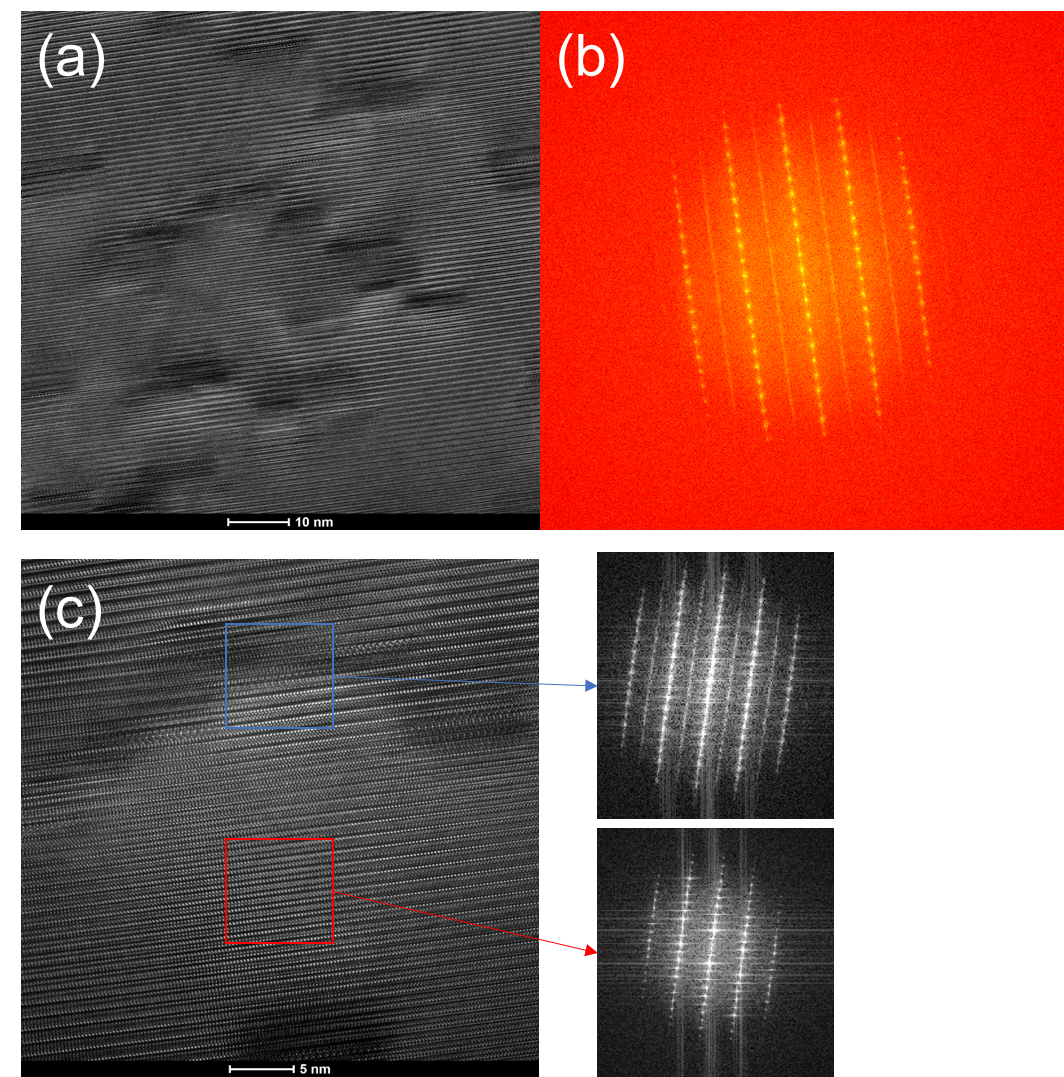


Supplementary Figure 1: the origin of the extra peaks and streaking. (a) the HREM image and (b) its fast Fourier transformation (FFT). (c) The FFT from two regions in the HREM image showing the emergence of the extra peaks at the polar nanoregion.


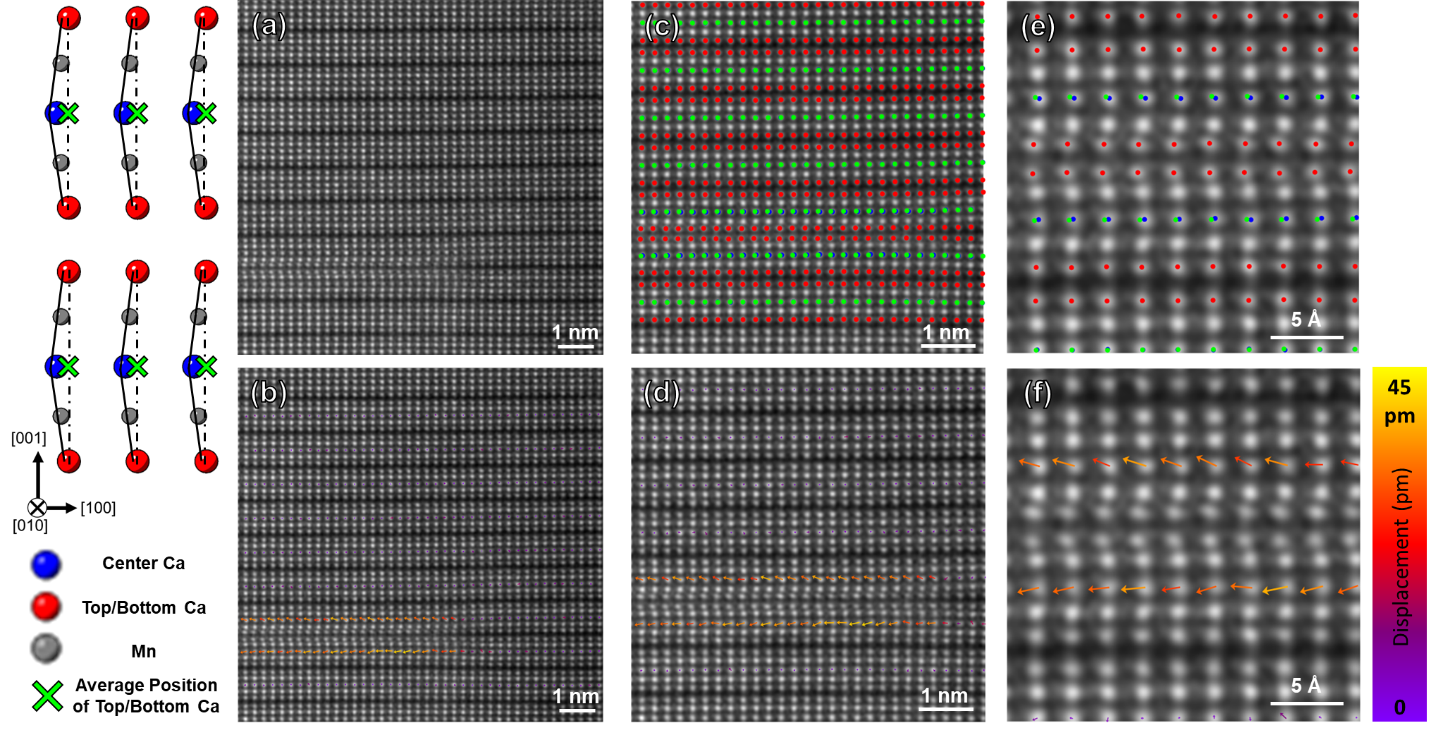


Supplementary Figure 2: Ca/Sr atom displacement measurement method on the a-type db-PNRs. (a) ADF-STEM image near the a-type db-PNRs. (b) the displacement vector map. (c) magnified ADF-STEM image with overlaying the atom positions for the center Ca/Sr atoms in the double perovskite blocks (green) and top&bottom Ca/Sr atoms (red). (d) the vector map in the same region as (c). (e) magnified ADF-STEM image near the a-type db-PNR with red, green and blue dots indicating the position for top&bottom, center and averaged position of top&bottom Ca/Sr atoms. (f) the corresponding vector map for (e).


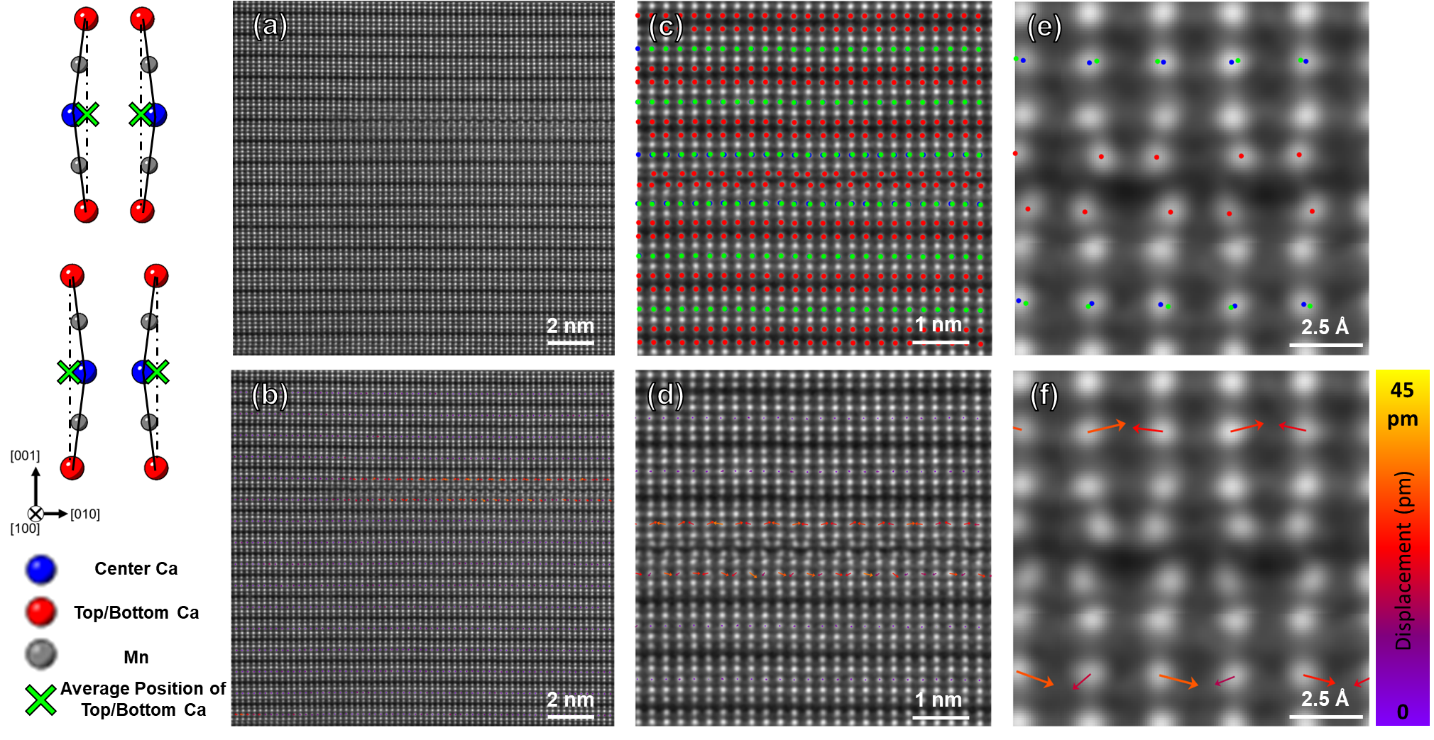


Supplementary Figure 3: Ca/Sr atom displacement measurement method on the b-type db-PNRs. (a) ADF-STEM image near the a-type db-PNRs. (b) the displacement vector map. (c) magnified ADF-STEM image with overlaying the atom positions for the center Ca/Sr atoms in the double perovskite blocks (green) and top&bottom Ca/Sr atoms (red). (d) the vector map in the same region as (c). (e) magnified ADF-STEM image near the b-type db-PNR with red, green and blue dots indicating the position for top&bottom, center and averaged position of top&bottom Ca/Sr atoms. (f) the corresponding vector map for (e).


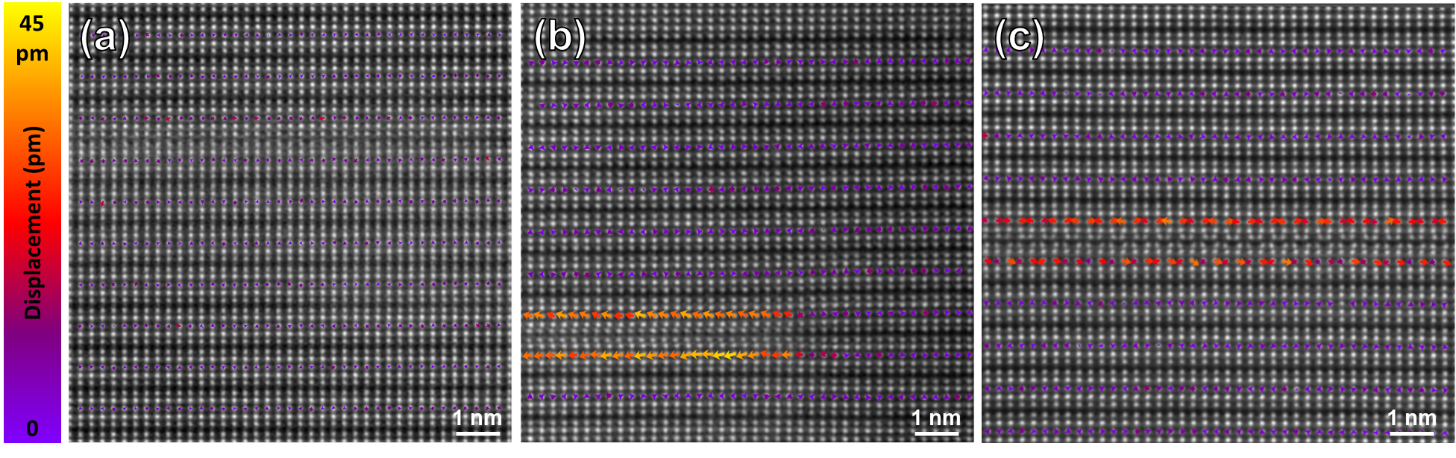


Supplementary Figure 4: Ca atom displacement measurement on (a) non-polar, (b) a-type and (c) b-type polar nanoregions respectively.


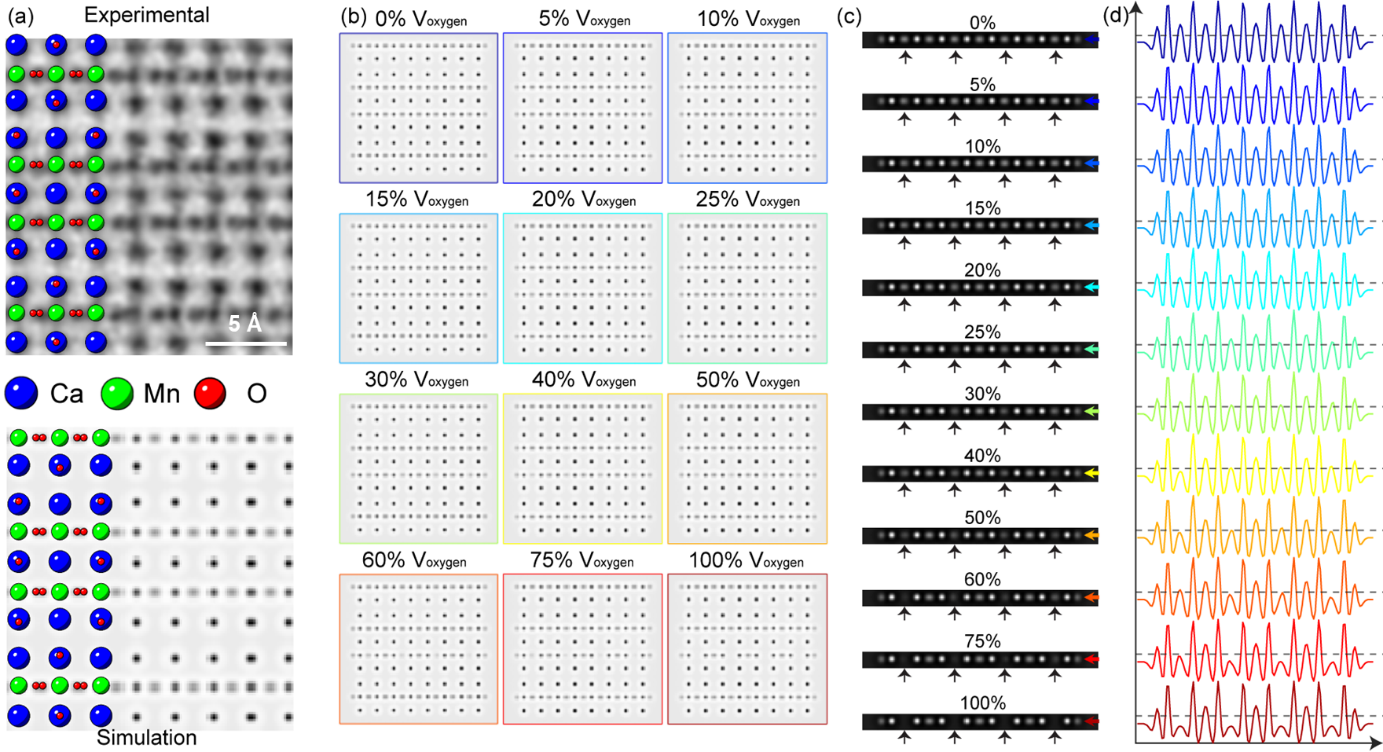


Supplementary Figure 5: annular bright field (ABF) STEM simulation for understanding the intensity modulation with the presence of the oxygen vacancies at different concentrations. (a) the experimental and simulated ABF-STEM image with the crystal model of Ca_3_Mn_2_O_7_ (*A*caa) superimposed. (c) the simulated ABF-STEM images using the crystal model with different percentage of oxygen vacancies randomly distributed in a row of oxygen columns. The columns potentially containing the oxygen vacancies are marked with black arrows. The numbers on top indicate the percentage of the oxygen vacancies. (d) the cropped image (contrast inversed) from the ABF-STEM image highlighting the contrast change. (e) the averaged intensity profile extract from (d). The dashed lines indicate the halved intensity of the oxygen column in the pristine crystal model with no vacancies. The column intensity consistently drops below the halved intensity lines if the vacancy percentage is over 40%.


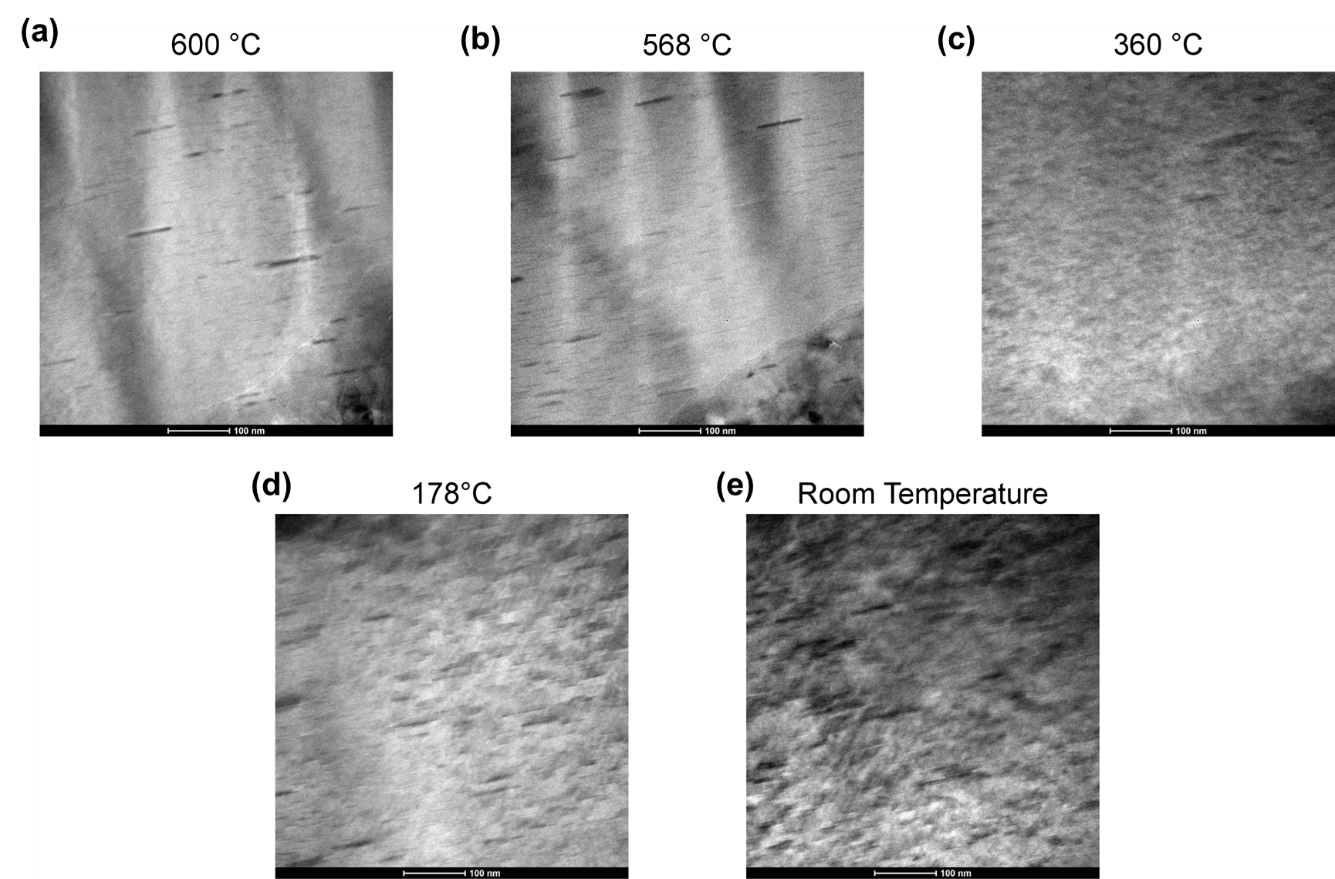


Supplementary Figure 6: The TEM micrographs of the morphology of the CSMO sample at different temperatures during the cooling process of the in-situ heating experiment taken from [100] zone axis. The TEM images are acquired at the temperature of (a) 600 °C, (b) 568 °C, (c) 360 °C, (d) 178 °C, and (e) 25 °C, respectively. The white scale bar is 100 nm. The TEM images are acquired without inserting the objective aperture and at a slight defocus to enhance the contrast.


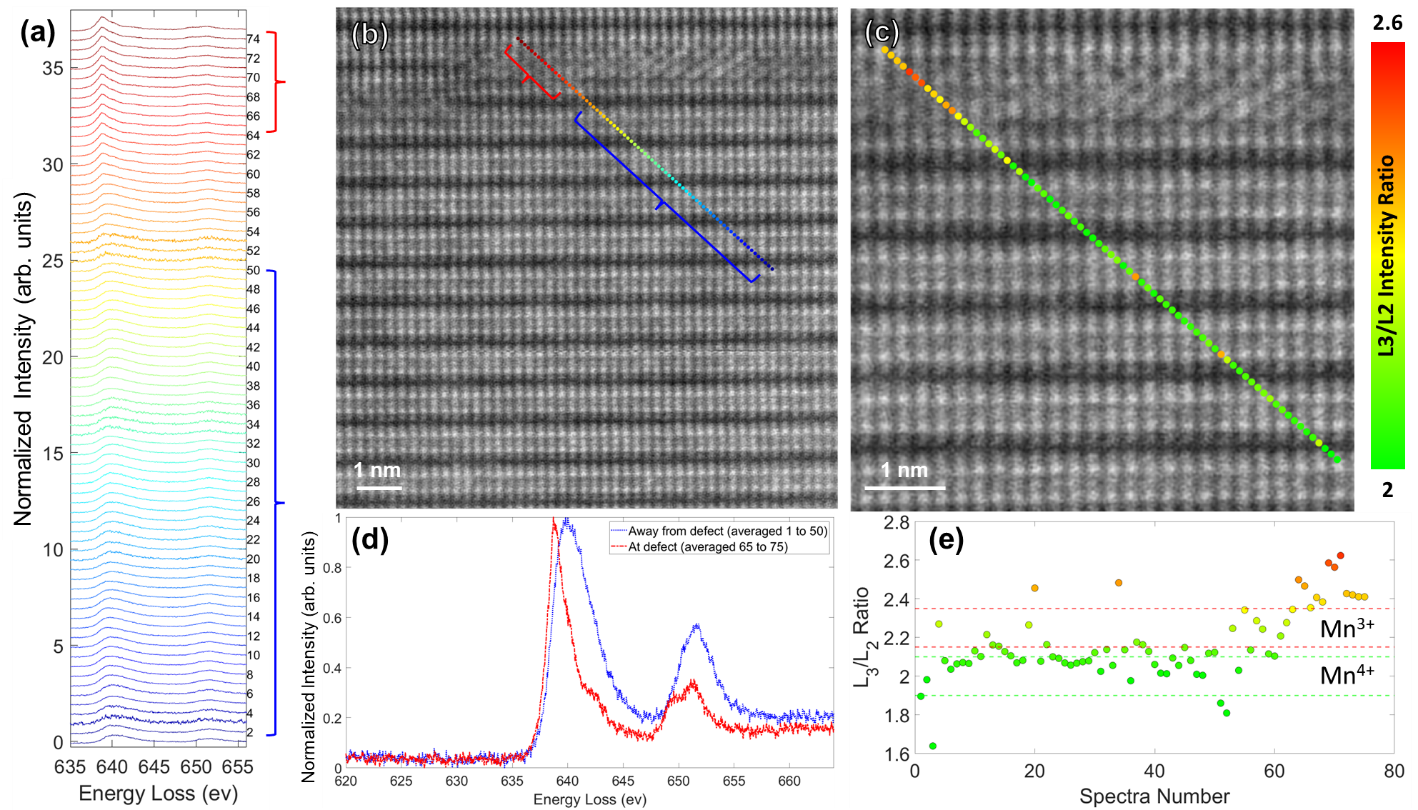


Supplementary Figure 7: Results of EELS Mn L edge L_3_/L_2_ ratio fitting from a line scan data containing spectra from both polar and non-polar regions. (a) The EELS spectra in the line scan data, with the corresponding probe positions plotted in (b). (c) the L_3_/L_2_ intensity ratio calculated from the spectra. The colors indicate the value of the intensity ratio. (d) the averaged spectra extracted from the polar nanoregions. (e) The L_3_/L_2_ ratio for EELS line scan data and the range of the intensity ratio for the Mn^3+^ and Mn^4+^.


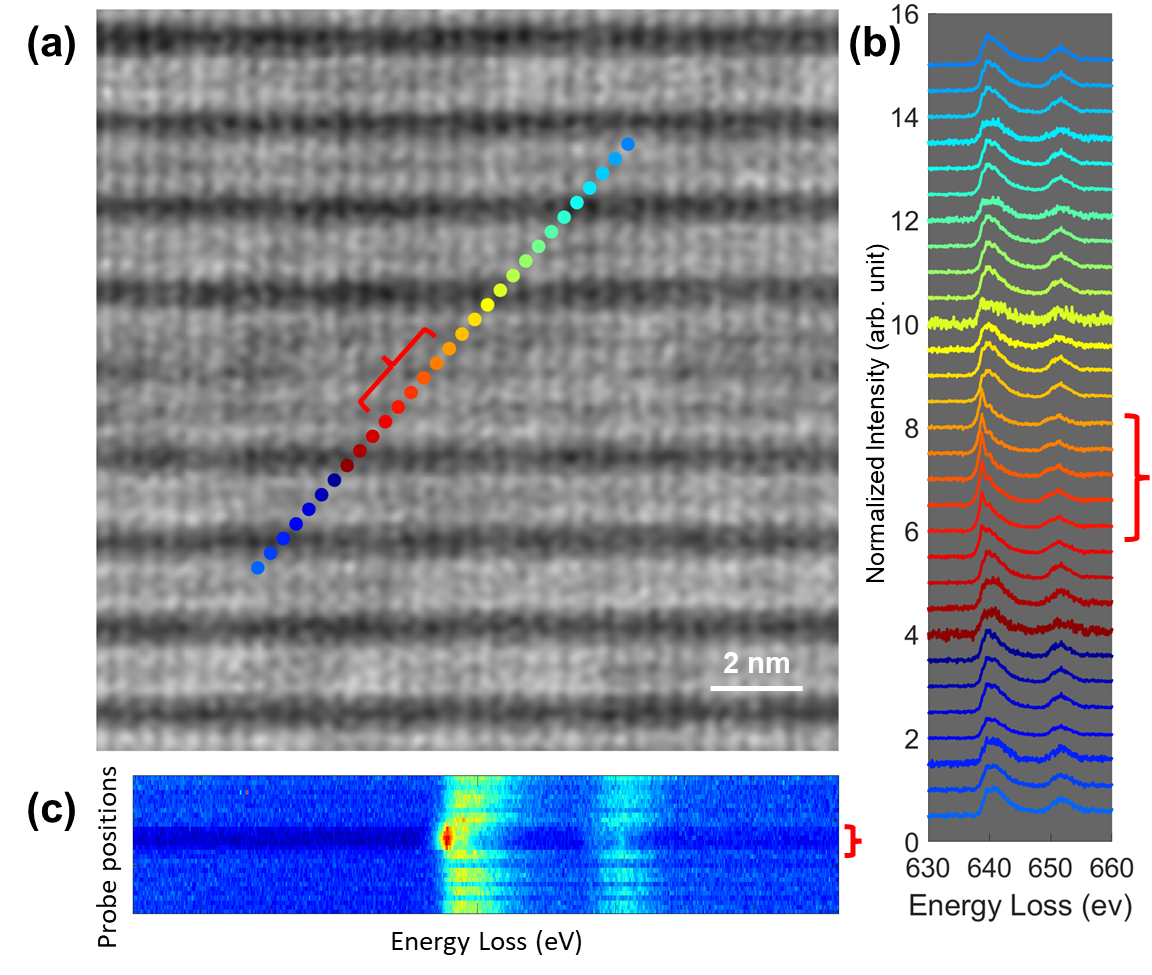


Supplementary Figure 8: Line-scan mono-chromated EELS spectra on the polar nanoregions. (a) the probe positions and (b) the energy loss near edge structure (ELNES) of Mn L edges. The ELNES change can be observed near the polar nanoregions. (c) the intensity plot of the EELS line scan data. The intensity is more intense near the polar nanoregions.


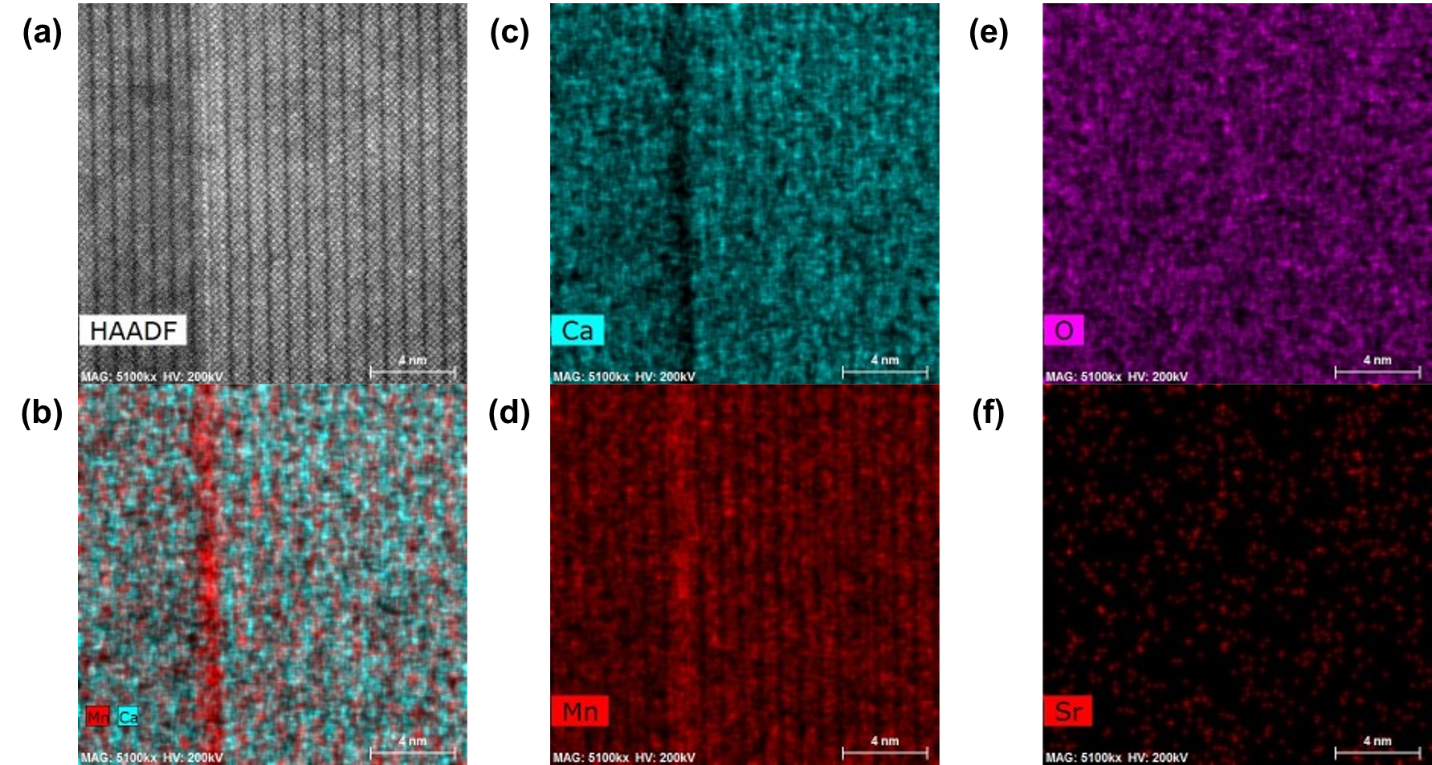


Supplementary Figure 9: Results the EDX elemental mapping performed with STEM around the double-bilayer polar nanoregions. (a) HAADF-STEM image. (b) Ca and Mn EDX mapping. A deficiency of (c) Ca and a surplus of (d) Mn can be observed, while (e) O and (f) Sr do not show clear differences with the crystal matrix.

Supplementary Figure 10: (a-h) Symmetry-distinct Mn-dopant configurations for Ca_2.5_Mn_2.5_O_7_. Blue, green, magenta, and red balls represent calcium, Mn^4+^, Mn^2+^, and oxygen atoms, respectively. The Mn-O-Mn bond angles shown are obtained from DFT+U-relaxed structures. The total energy of each structure computed from DFT+U is reported in Table S1. We find that configuration (a) has the lowest energy, and thus focus on this configuration in the main text. We note that we only consider one configuration with a Mn-dopant located on the A-site in the center of the perovskite bilayer (h) because this is much higher in energy than configurations with all Mn-dopants in the rocksalt layer (see Table S2).

Supplementary Figure 11: (a-e) Symmetry-distinct Mn-dopant configurations for Ca_2.25_Mn_2.75_O_7_. Blue, green, magenta, and red balls represent calcium, Mn^4+^, Mn^2+^, and oxygen atoms, respectively. The Mn-O-Mn bond angles shown are obtained from DFT+U-relaxed structures. The total energy of each structure computed from DFT+U is reported in Table S3. We find that configuration (a) has the lowest energy, and thus focus on this configuration in the main text.


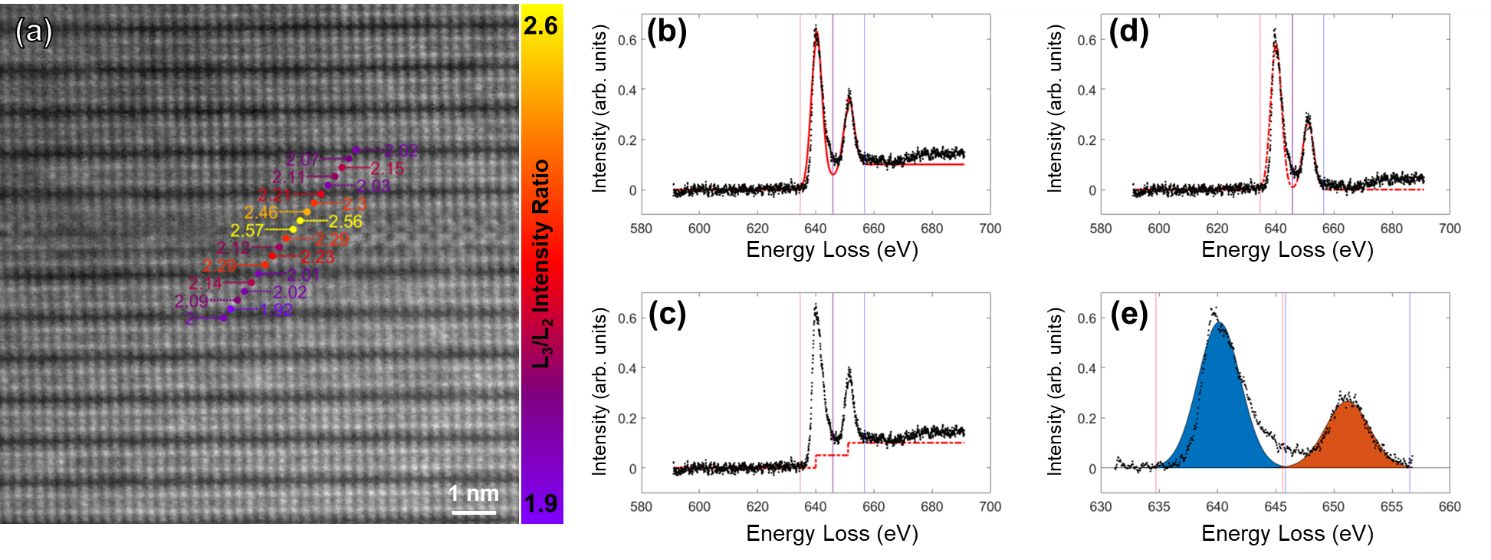


Supplementary Figure 12: Method for EELS Mn L edge L_3_/L_2_ ratio fitting. (a) the L_3_/L_2_ intensity ratio plot of the EELS line scan data. (b) The background intensity is fitted with the power law and (c) a step function and then (d) subtracted. (e) The peak position is then fitted with two gaussian peaks and the intensity of two peaks are integrated around the L_3_ and L_2_ peak positions.

Supplementary Table 1: Lattice parameters of Ca_3-x_Mn_2+x_O_7_ structures with symmetries *A*2_1_am and *A*caa obtained from DFT+U structural relaxations. For structures with x>0, the lattice parameters are given for the structure with the lowest energy dopant configuration.

| Material | x | Lattice parameter, *A*2_1_am (Å) | | | Lattice parameter, *A*caa (Å) | | |
| --- | --- | --- | --- | --- | --- | --- | --- |
|  |  | *a* | *b* | *c* | *a* | *b* | *c* |
| Ca_3_Mn_2_O_7_ | 0 | 5.22 | 5.22 | 19.23 | 5.17 | 5.17 | 19.56 |
| Ca_2.75_Mn_2.25_O_7_ | 0.25 | 5.24 | 5.22 | 19.09 | 5.17 | 5.17 | 19.46 |
| Ca_2.5_Mn_2.5_O_7_ | 0.50 | 5.24 | 5.21 | 18.93 | 5.15 | 5.15 | 19.38 |
| Ca_2.25_Mn_2.75_O_7_ | 0.75 | 5.24 | 5.20 | 18.82 | 5.15 | 5.15 | 19.30 |
| Ca_2_Mn_3_O_7_ | 1.00 | 5.25 | 5.20 | 18.63 | 5.14 | 5.14 | 19.18 |
| CaMn_4_O_7_ | 2.00 | 5.28 | 5.17 | 18.03 | 5.12 | 5.11 | 18.81 |

Supplementary Table 2: Energy of Ca_2.5_Mn_2.5_O_7_ in the *A*2_1_am phase with different dopant configurations from DFT+U calculations. Structure (a) with all dopants in same rocksalt layer has the lowest energy. The labelling of the configurations (a-h) corresponds to the structures shown in Figure S10. Energies are reported relative to that of configuration (a), which is set to 0 meV/f.u..

| Dopant configuration | Energy (meV/f.u.) |
| --- | --- |
| (a) Ca_2.5_Mn_2.5_O_7_ | 0.00 |
| (b) Ca_2.5_Mn_2.5_O_7_ | 42.82 |
| (c) Ca_2.5_Mn_2.5_O_7_ | 32.93 |
| (d) Ca_2.5_Mn_2.5_O_7_ | 36.98 |
| (e) Ca_2.5_Mn_2.5_O_7_ | 45.82 |
| (f) Ca_2.5_Mn_2.5_O_7_ | 42.10 |
| (g) Ca_2.5_Mn_2.5_O_7_ | 27.69 |
| (h) Ca_2.5_Mn_2.5_O_7_ | 81.38 |

Supplementary Table 3: Energy of Ca_2.25_Mn_2.75_O_7_ in the *A*2_1_am phase with different dopant configurations from DFT+U calculations. Structure (a) with all dopants in same rocksalt layer has the lowest energy. The labelling of the configurations (a-e) corresponds to the structures shown in Figure S11. Energies are reported relative to that of configuration (a), which is set to 0 meV/f.u..

| Dopant configuration | Energy (meV/f.u.) |
| --- | --- |
| (a) Ca_2.25_Mn_2.75_O_7_ | 0.00 |
| (b) Ca_2.25_Mn_2.75_O_7_ | 63.66 |
| (c) Ca_2.25_Mn_2.75_O_7_ | 53.86 |
| (d) Ca_2.25_Mn_2.75_O_7_ | 57.64 |
| (e) Ca_2.25_Mn_2.75_O_7_ | 50.21 |
